# Dynamic Interaction between STLV-1 Proviral Load and T-Cell Response during Chronic Infection and after Immunosuppression in Non-Human Primates

**DOI:** 10.1371/journal.pone.0006050

**Published:** 2009-06-25

**Authors:** Sandrine Souquière, Augustin Mouinga-Ondemé, Maria Makuwa, Olivier Hermine, Mirdad Kazanji

**Affiliations:** 1 Unité de Rétrovirologie, Centre International de Recherches Médicales de Franceville (CIRMF), Franceville, Gabon; 2 Department of Hematology, Necker Hospital, Paris, France; 3 Réseau International des Instituts Pasteur, Institut Pasteur, Paris, France; University of Sao Paulo, Brazil

## Abstract

We used mandrills (*Mandrillus sphinx*) naturally infected with simian T-cell leukemia virus type 1 (STLV-1) as a model for evaluating the influence of natural STLV-1 infection on the dynamics and evolution of the immune system during chronic infection. Furthermore, in order to evaluate the role of the immune system in controlling the infection during latency, we induced immunosuppression in the infected monkeys. We first showed that the STLV-1 proviral load was higher in males than in females and increased significantly with the duration of infection: mandrills infected for 10–6 years had a significantly higher proviral load than those infected for 2–4 years. Curiously, this observation was associated with a clear reduction in CD4+ T-cell number with age. We also found that the percentage of CD4^+^ T cells co-expressing the activation marker HLA-DR and the mean percentage of CD25^+^ in CD4^+^ and CD8^+^ T cells were significantly higher in infected than in uninfected animals. Furthermore, the STLV-1 proviral load correlated positively with T-cell activation but not with the frequency of T cells secreting interferon γ in response to *Tax* peptides. Lastly, we showed that, during immunosuppression in infected monkeys, the percentages of CD8^+^ T cells expressing HLA-DR^+^ and of CD4^+^ T cells expressing the proliferation marker Ki67 decreased significantly, although the percentage of CD8^+^ T cells expressing HLA-DR^+^ and Ki67 increased significantly by the end of treatment. Interestingly, the proviral load increased significantly after immunosuppression in the monkey with the highest load. Our study demonstrates that mandrills naturally infected with STLV-1 could be a suitable model for studying the relations between host and virus. Further studies are needed to determine whether the different compartments of the immune response during infection induce the long latency by controlling viral replication over time. Such studies would provide important information for the development of immune-based therapeutic strategies.

## Introduction

Human T-cell lymphotropic virus type 1 (HTLV-1), the causative agent of adult T-cell leukemia/lymphoma (ATLL) [1] and of tropical spastic paraparesis/HTLV-1-associated myelopathy (TSP/HAM) [2], has also been implicated in pediatric infectious dermatitis [3], uveitis [4], arthropathy [5] and polymyositis [6].

Simian T cell leukemia virus (STLV–1), the simian counterpart of HTLV-1, naturally infects Old World monkeys and shares virologic, immunologic, molecular, and pathologic features with HTLV-1 [7]–[9]. The phylogenic relations of most known subtypes indicate that STLV-1 is the simian ancestor of HTLV-1, the latter arising by transmission from multiple nonhuman primates to humans [10]–[14]. In naturally infected monkeys, STLV-1 causes diseases similar to those induced by HTLV-1 in humans, ATLL-like pathological features occurring in a minority of individuals after a long latency [15]–[18].

In Africa, STLV-1 infection has been detected in both species of great ape (*Pan* and *Gorilla*) as well as in the Old World monkey family Cercopithecidae [9]. The mandrill (*Mandrillus sphinx*), with a geographic distribution restricted to the tropical forests of Cameroon, Equatorial Guinea, Gabon, and southern Congo [11], [19], is subject to infection with an STLV-1 subtype closely related to HTLV-1. The first cases of natural STLV infection in mandrills (STLVmnd) were detected during a retrospective serological survey at the Primate Centre of the International Centre for Medical Research (CIRMF) in Gabon. Two males were STLV-1-seropositive on their arrival at CIRMF, strongly supporting the existence of STLV infection in the wild. Natural transmission of the virus has been monitored since the breeding colony was created in 1983 [20]. The first genetic studies of STLVmnd indicated the presence of two genetically distinct strains [19], intracolony transmission occurring mainly through male–male aggression [20], [21]. It has also been reported that STLV from mandrills is closely related to HTLV-1. The possibility of cross-species transmission of STLV-1 to humans is supported by the fact that the STLVmnd viruses so far characterized are genetically similar to HTLV-1 subtype D and F viruses isolated from people living in central Africa [11], [19]. Moreover, African HTLV-1 and STLV-1 cannot be separated into distinct phylogenetic lineages on the basis of their species of origin, but rather on the geographic origin of their hosts. All this suggests that STLV infection of mandrills could serve as a model of human HTLV infection. Although the phylogenetics of STLV-1 infection of mandrills are well documented, few data are available on the virologic or molecular determinants of natural STLV-1 infection in this host species. Moreover, little is known about the initial and latent phases of STLV-1/HTLV-1 infection in terms of proviral load, reservoir cells, viral expression, the pattern of viral integration (clonality), the initial target cells, and the role of cytotoxic T-cell responses during natural infection in nonhuman primates. We therefore examined immunological and virological patterns in a large group of naturally STLV-1-infected mandrills, including T-cell subsets, proviral load, and STLV-specific T-cell responses. Furthermore, to evaluate the role of the immune system in controlling STLV-1 infection, we induced immunosuppression in naturally infected mandrills and evaluated the dynamics of immunologic and virologic parameters during and after treatment.

## Results

### High STLV-1 prevalence and high proviral load in naturally infected mandrills

Sera from 142 mandrills (58 males, 84 females; mean age, 10.7 years) were tested by ELISA for the presence of anti-STLV-1 antibodies. Positive samples were confirmed by western blotting. As shown in Table 1, 19 mandrills (13.4%), all with complete western blot profiles, had antibodies against HTLV-1. The prevalence of STLV-1 infection increased significantly with age (*p* = 0.001), from 0% at 1–4 years to 12.9% at 5–10 years, 18.1% at 11–15 years, and 33.3% at >16 years. More males than females were infected (17.2% vs 10.7%). No mandrills under 4 years of age were infected. The prevalence remained stable with age in females but increased with age in males, reaching 87.5% at >16 years.

**Table 1:** Distribution of STLV-1 infection in our semi-free-ranging colony of 142 mandrills by age group and sex.

Age	Male		Female		Total	
	No. positive/tested	%	No. positive/tested	%	Total	%
1–4	0/16	0	0/21	0	0/37	0
5–10	2/30	6.6	6/32	18.8	8/62	12.9
11–15	1/4	25	3/18	16.7	4/22	18.1
>16	7/8	87.5	0/13	0	7/21	33.3
**Total**	**10/58**	**17.2**	**9/84**	**10.7**	**19/142**	**13.4**

**Figure 1:**
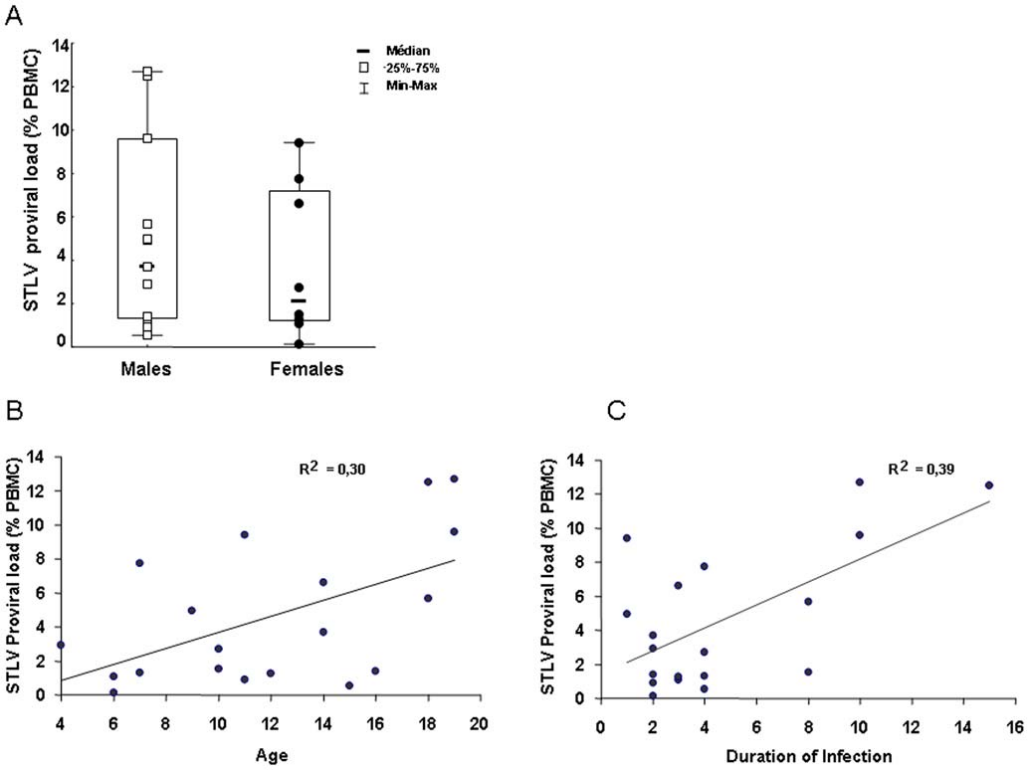
STLV-1 proviral load as detected by real-time PCR in 19 mandrills, expressed as copy numbers of provirus per 100 PBMC. (A) Proviral load distribution in male and female monkeys. (B) Correlation of STLV-1 proviral load with age of monkeys. (C) Correlation of STLV-1 proviral load with duration of STLV-1 infection.

STLV-1 proviral load was determined by real-time PCR for 19 infected mandrills (11 males and 8 females). On average, 4.56% of peripheral blood mononuclear cells (PBMC) were infected (range, 0.54–12.7%); however, the mean proviral load was higher in males (5.11%) than in females (3.81%) (Figure 1A) and increased with age (Figure 1B). Furthermore, the proviral load increased significantly with the duration of infection (Figure 1C): mandrills infected for 8–15 years had significantly higher proviral loads than those infected for 2–4 years (mean, 8.4±4.8 versus 3.2±2.9).

### Evaluation of T-cell subsets and influence of STLV-1 infection on T-cell response

**Figure 2:**
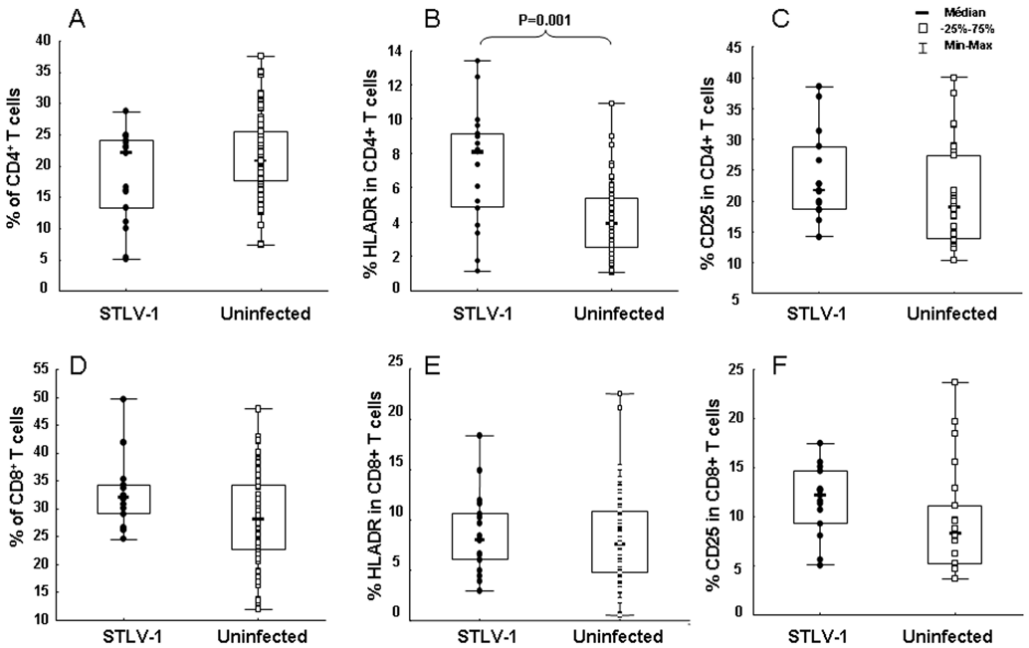
Comparisons of T-cell subsets in blood from 17 out of 19 mandrills infected with STLV (•) and 59 uninfected mandrills (□). (A) Percentages of CD3^+^CD4^+^ cells. (B) Percentages of HLA-DR in CD4^+^ T cells. (C) Percentages of CD25^+^ in CD4^+^ T cells. (D) Percentages of CD3^+^CD8^+^ cells. (E) Percentages of HLA-DR in CD8^+^ T cells. (F) Percentages of CD25^+^ in CD8^+^ T cells. Statistical analyses for each T-cell subset in the two groups of mandrills were performed with the Mann-Witney test. The only significant difference was in the percentage of HLADR in CD4^+^ T cells (*p* = 0.001).

In order to evaluate the role of STLV-1 infection in the evolution of the cellular immune response, the distribution of T-cell subsets defined by the markers CD4, CD8, HLA-DR, and CD25 were compared in 17 STLV-1-infected (two infected mandrills died of old age) and 59 uninfected animals. As shown in Figure 2A and 2D, the percentages of CD4^+^ and CD8^+^ T cells were similar. In contrast, the percentage of CD4^+^ T cells co-expressing the activation marker HLA-DR was significantly higher in infected than in uninfected animals (*p* = 0.001) (Figure 2B), even though no change was noted in the percentage of HLA-DR markers in CD8^+^ T cells (Figure 2E). Furthermore, the mean percentages of CD25^+^ in CD4^+^ and CD8^+^ T cells (24.0% and 11.7%, respectively) were higher in infected than in uninfected animals (20.8% and 9.6%, respectively); this difference was not statistically significant (Figure 2C and 2F).

**Figure 3:**
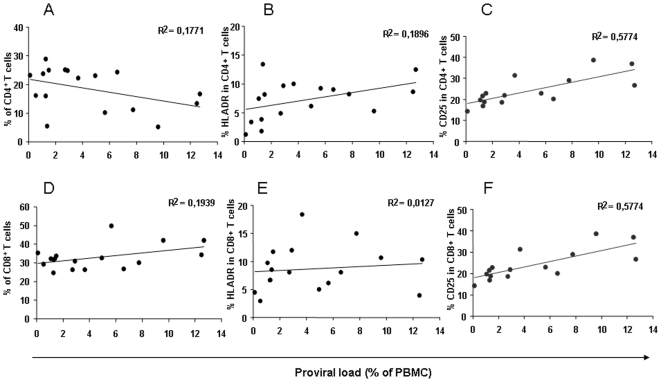
Evolution of T-cell subsets in the 17 STLV-infected mandrills, correlated with their respective proviral load. (A) Percentages of CD3^+^CD4^+^ cells. (B) Percentages of HLA-DR in CD4^+^ T cells. (C) Percentages of CD25 in CD4^+^ T cells. (D) Percentages of CD3^+^CD8^+^ cells. (E) Percentages of HLA-DR in CD8^+^ T cells. (F) Percentages of CD25 in CD8^+^ T cells. Regression curves are shown as black lines.

The percentages of T-cell subsets were then correlated with the proviral load. As seen in Figure 3, the percentage of CD4+ T cells correlated negatively with the STLV-1 proviral load, while the percentage of CD8^+^ T cells showed a positive correlation (Figure 3A and 3D). A positive correlation was also found between the proviral load and the percentages of HLA-DR^+^ in CD4^+^ T cells and CD25^+^ in CD4^+^ T cells and CD8^+^ T cells (Figure 3B, C, and F). No correlation was found between the STLV-1 proviral load and the percentage of CD8^+^ cells expressing the activation marker HLA-DR^+^ (Figure 3E).

### STLV-1-specific cellular immune responses in chronically infected mandrills

**Figure 4:**
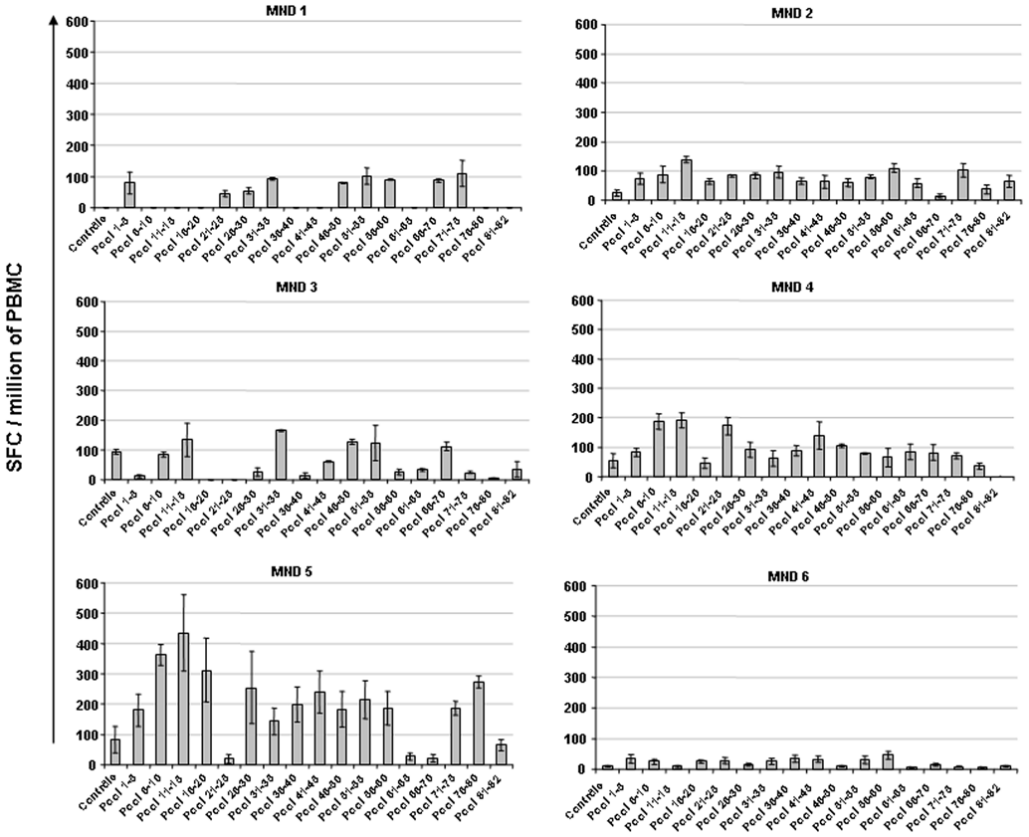
Frequency of specific IFNγ-secreting T cells in six naturally STLV-infected mandrills. PBMC were cultured in the presence of 17 different pools (containing five peptides) at 10 µmol/ml, overlapping the entire *Tax* gene of STLVmnd (see Table 2). IFNγ-releasing cells were evaluated with the ELISPOT assay. Background levels were measured in wells containing irrelevant peptides or medium only. The frequency of responding cells is presented after subtraction of background spots obtained with medium alone. Each bar represents the mean number of IFNγ spots in triplicate wells per million PBMC.

The frequency of T cells secreting interferon (IFN)-γ in response to 82 overlapping peptides covering the totality of the *Tax* gene (Table 2) was evaluated by ELISPOT in the six chronically STLV-1-infected mandrills and in one uninfected monkey. As shown in Figure 4, T cells from all six mandrills secreted IFN-γ in the presence of at least one pool of peptides. All the peptide pools were able to induce IFN-γ secretion in the STLV-1-infected monkeys but not in the uninfected control, and none was found to be more immunogenic than the others. No correlation was found between STLV proviral load and the frequency of STLV-specific cellular immune responses (data not shown). It is noteworthy, however, that the strongest cellular immune response was found in an animal (#MND 5) with a low proviral load (1.3%). Therefore, to evaluate the role of the cellular immune response in controlling STLV-1, we investigated the effect of immunosuppressive treatment on the dynamics of the STLV proviral load and on the distribution of T-cell subsets.

**Table 2:** Pools of 82 overlapping peptides (12-mers) covering the entire STLV-1 Tax protein, used for stimulation in vitro of PBMCs from STLV-1-infected mandrills.

Pool	Peptide No	Sequences	Pool	Peptide No	Sequences
Pool 1–5	1	LLFGYPVYVFGD	Pool 41–45	41	HPGQLGAFLTNV
	2	YPVYVFGDCVQG		42	LGAFLTNVPYKR
	3	VFGDCVQGDWCP		43	LTNVPYKRMEEL
	4	CVQGDWCPISGG		44	PYKRMEELLYKI
	5	DWCPISGGLCSA		45	MEELLYKISLTT
Pool 6–10	6	ISGGLCSARLHR	Pool 46–50	46	LYKISLTTGALI
	7	LCSARLHRHALL		47	SLTTGALIILPE
	8	RLHRHALLATCP		48	GALIILPEDCLP
	9	HALLATCPEHQI		49	ILPEDCLPTTLF
	10	ATCPEHQITWDP		50	DCLPTTLFQPAR
Pool 11–15	11	EHQITWDPIDGR	Pool 51–55	51	TTLFQPARAPAT
	12	TWDPIDGRVIGS		52	QPARAPATLTAW
	13	IDGRVIGSALQF		53	APATLTAWQNGL
	14	VIGSALQFLIPR		54	LTAWQNGLLPFH
	15	ALQFLIPRLPSF		55	QNGLLPFHSTLT
Pool 16–20	16	LIPRLPSFPTQR	Pool 56–60	56	LPFHSTLTTPGL
	17	LPSFPTQRTSKT		57	STLTTPGLIWTF
	18	PTQRTSKTLKVL		58	LIWTFTDGTPMI
	19	TLKVLTPPTTHT		59	FTDGTPMISGPC
	20	LTPPTTHTTPNI		60	TPMISGPCPKDG
Pool 21–25	21	TTHTTPNIPPSF	Pool 61–65	61	SGPCPKDGQPSL
	22	TPNIPPSFLQAM		62	PKDGQPSLVLQS
	23	PPSFLQAMRKYS		63	QPSLVLQSSSFI
	24	LQAMRKYSPFRN		64	VLQSSSFIFHKF
	25	RKYSPFRNGYME		65	SSFIFHKFQTKA
Pool 26–30	26	PFRNGYMEPTLG	Pool 66–70	66	FHKFQTKAYHPS
	27	GYMEPTLGQHLP		67	QTKAYHPSFLLS
	28	PTLGQHLPTLSF		68	YHPSFLLSHGLI
	29	QHLPTLSFPDPG		69	FLLSHGLIQYSS
	30	TLSFPDPGLRPQ		70	HGLIQYSSFHNL
Pool 31–35	31	PDPGLRPQNLYT	Pool 71–75	71	QYSSFHNLHLLF
	32	LRPQNLYTLWGS		72	FHNLHLLFEEYT
	33	NLYTLWGSSVVC		73	HLLFEEYTNIPI
	34	LWGSSVVCMYLY		74	EEYTNIPISLLF
	35	SVVCMYLYQLSP		75	NIPISLLFNEKE
Pool 36–40	36	MYLYQLSPPITW	Pool 76–80	76	SLLFNEKEANDT
	37	QLSPPITWPLLP		77	LFNEKEANDTDH
	38	SPPITWPLLPHV		78	NDTDHENGISPG
	39	LLPHVIFCHPGQ		79	HENGISPGGIEP
	40	VIFCHPGQLGAF		80	ISPGGIEPPSEK
			Pool 81–82	81	GIEPPSEKHFRE
Control	83	GILGFVFTL		82	PPSEKHFRETEV

Peptides were diluted to obtain 10 µg/ml final concentration and pooled in groups of five. Peptide #83, located on the matrix protein of influenza A virus, was used as control.

### Effect of immunosuppressive treatment in mandrills chronically infected with STLV-1

Two groups of three mandrills were selected on the basis of their proviral burden, with low, medium and high burdens in each group. The first group was immunosuppressed with tacrolimus, whereas the second group received phosphate-buffered saline as a control. Both the proviral load and the CD4, CD8, HLA-DR, and Ki67 T cell populations were evaluated before (week –2, week –1, and day 0), during (weeks +1, +2, and +3) and after treatment (weeks +4, +5, +6, +7, and +8).

**Figure 5:**
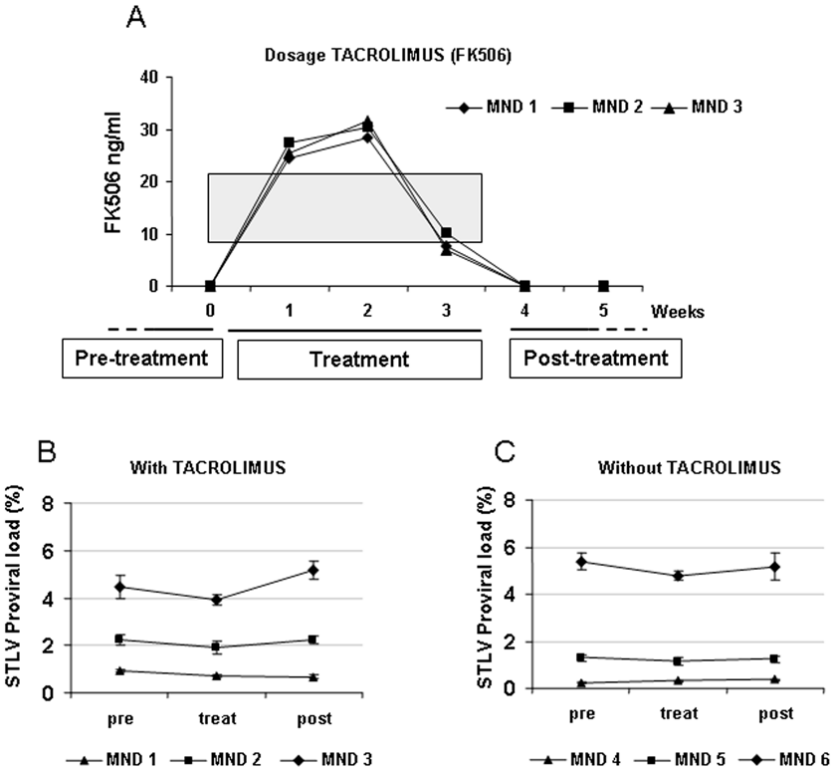
Pharmacokinetics and virological data before (pre), during (treat) and after (post) immunusuppressive therapy. (A) Pharmacokinetics of tacrolimus in blood over 5 weeks in three STLV-1-infected mandrills. The grey zone indicates the effective therapeutic dose in humans. Immunological and virological data were analysed at different phases of the experiment: pretreatment corresponds to weeks –2, –1, and 0 (pre); treatment corresponds to weeks 1, 2, and 3 (treat); post-treatment corresponds to weeks 4, 5, 6, 7, and 8 (post). (B) Kinetics of STLV proviral load during immunusuppressive therapy. Each value represents the mean±SD of measurements in triplicate for the three phases of the experiment. (C) Kinetics of STLV proviral load in the three control animals.

No effect on the biochemical markers usually seen during immunosuppression in humans was found (data not shown). One week after immunosuppression, tacrolimus was detected in the blood of treated monkeys within the therapeutic range used in humans (Figure 5A).

Proviral load was expressed as the mean value for each animal before, during, and after therapy. As seen in Figure 5B and 5C, there was no significant variation in the proviral load in control or treated mandrills with low or medium proviral loads, but the proviral load increased significantly in the mandrill with the highest proviral load (MND 3) after tacrolimus administration (Figure 5B).

**Figure 6:**
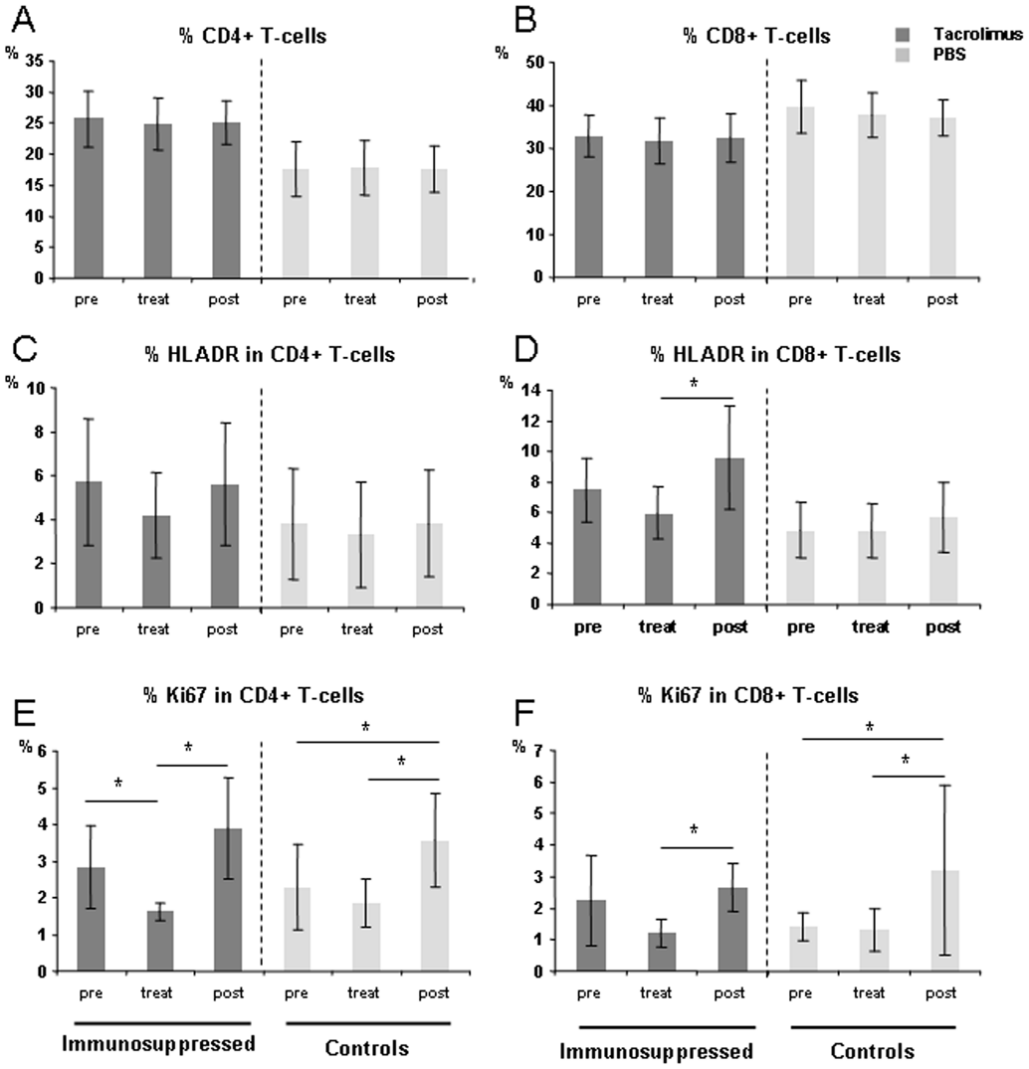
Effect of tacrolimus on T-cell subsets as measured before (pre), during (treat) and after treatment (post). Dark-grey bars represent mean values±SD for the three mandrills treated with tacrolimus. Means±SD for the three control mandrills are shown as pale-grey bars. (A) Percentages of CD4^+^ T cells. (B) Percentages of CD8^+^ T cells. (C) Expression of HLA-DR in CD4^+^ T cells and (D) in CD8^+^ T cells. (E) Expression of Ki67 in CD4^+^ T cells and (F) in CD8^+^ T cells. *, *p*<0.01 as determined with the Mann-Whitney *U* test.

The mean numbers of T-cell subsets were measured in all animals in each group before, during, and after tacrolimus treatment. Tacrolimus did not affect the percentages of CD4^+^ or CD8^+^ T cells (Figure 6A and 6B) or the disruption of naive and memory T cells (data not shown). In contrast, the percentage CD8^+^ expression of HLA-DR+ decreased significantly during therapy (*p* = 0.01) (Figure 6D), whereas the percentage of CD4+ T cells expressing the proliferation marker Ki67 decreased significantly during treatment (*p* = 0.01) but increased significantly after the end of treatment (*p*<0.001), as did the percentage of CD8^+^ T cells expressing Ki67 (*p*<0.001) (Figure 6E and 6F). The control groups showed no statistically significant change in the percentages of CD4^+^ and CD8^+^ cells expressing HLA-DR during or after treatment (Figure 6C and 6D).

## Discussion

Our study provides the first in-depth virological and immunological characterization of natural STLV-1 infection in a nonhuman primate. We further evaluated the influence of this natural infection on the dynamics and evolution of the immune system during chronic infection and after the induction of immunosuppression in the infected monkeys.

When the mandrill colony was created in 1983, only two founder males were infected with STLV-1. Phylogenetic studies have since shown that they were infected with subtypes D and F [19]. A retrospective study of the same colony showed that in 1995 eight males were STLV-1-seropositive. The suspected mode of transmission was through bites inflicted during fights for dominance [21]. No females were infected at that time. The data reported here show that the prevalence is now high in males and increases markedly with age. We also show that females are now infected, but at a lower prevalence than in males, even among the oldest females. Transmission during fights cannot be ruled out, but sexual transmission, as in humans, should be considered. No cases of infection were detected among juveniles (<4 years), ruling out transmission *in utero* or during breastfeeding. Thus, the modes of STLV-1 transmission appear to be slightly different from those in humans, where the major mode of transmission is sexual or from mother to child during breastfeeding. It was reported previously that the rate of HTLV-1 transmission in humans, particularly from infected mothers to infants, increases significantly when women have a high HTLV-1 proviral load [22], [23]. Thus, even if the route of STLV-1 transmission in mandrills is different, the high proviral load in dominant male mandrills might explain the high rate of STLV-1 transmission in the colony. A recent study in a baboon colony showed that females were more frequently infected than males [24]. These results illustrate that different patterns of behavior of different nonhuman primates result in different profiles of virus circulation.

We also showed a wide range of STLV-1 proviral loads in PBMCs of infected monkeys. In some mandrills, the proviral load was very high (up to 12.7% of PBMCs), reaching levels usually associated with the onset of clinical manifestations in HTLV-1-infected humans, whereas the mandrills naturally infected with STLV-1 appeared healthy. Few reports have been made of the STLV proviral load in naturally infected monkeys [25]. Those authors reported a high STLV-1 proviral load in naturally infected Celebes macaques (*Macaca tonkeana*) without inflammatory disease. Nevertheless, HTLV-1-associated disease is rare in humans, with less than 5% infections after a long incubation period [26]. In order to demonstrate that STLV-1 can also induce inflammatory disease in its natural host, however, a long-term clinical survey of infected monkeys will be necessary.

Our study shows that proviral load is correlated positively with the duration of infection and thus with the age of the animals. No follow-up study of STLV-1-infected monkeys has previously been reported. In some studies in humans, however, there was no clear evidence of an association between proviral load and duration of infection [27]. It has been shown that the HTLV-1 proviral load is a strong predictor of disease progression, and patients with TSP/HAM or adult T-cell leukemia have a higher proviral load than asymptomatic HTLV-1 carriers [28], [29]. Furthermore, people infected with HTLV-1 develop associated diseases such as ATLL after long latency periods (30–40 years). It has been suggested previously that the development of associated disease in older individuals could be related to an immunosenescence phenomenon that leads to expansion and activation of HTLV-1-infected cells [30]. In order to clarify the relation between the increased level of STLV-1 in older monkeys and the role of the immune response during life, we evaluated the numbers of CD4^+^ and CD8^+^ T cells in STLV-1-infected and uninfected monkeys of different ages and observed clear reductions in the absolute numbers of both CD4^+^ and CD8^+^ T cells in older than in younger monkeys (Figure S1). Thus, the equilibrium between viral replication and the destruction and activation of CD4^+^ and CD8^+^ T cells might define the course of STLV-1 infection during the long latency. The loss of CD4^+^ T cells could induce a state of immunodeficiency in older individuals. These findings are in accordance with the observation that disequilibrium of the immune system with age can lead to uncontrolled virus expansion and thus to a high proviral load, which could lead further to disease progression. Each STLV-1-infected monkey will have to be followed-up to evaluate the dynamics of the STLV-1 proviral load and its correlation with various modifications in the immune response that could lead to disease progression after the long latency. These studies are under way.

In the present study, we found that the percentage of CD4^+^ T cells co-expressing the activation marker HLA-DR^+^ was higher in STLV-1-infected monkeys than in uninfected control animals. Furthermore, there was a significant correlation between activated CD4^+^CD25^+^ cells, CD8^+^CD25^+^ cells and proviral load, and primates with high proviral loads had elevated numbers of these cells. In humans, HTLV-1 leukemic cells have an activated helper/inducer T-cell phenotype, CD4^+^CD25^+^, which could be activated by the HTLV-1 Tax oncogene [31], [32], [33]. We showed recently that both CD4^+^ and CD8^+^ T cells are infected in STLV-1 infected mandrills [34]. Furthermore, a spontaneous T-cell leukaemia/lymphoma has been described in some African monkeys naturally infected with STLV-1 [16], [17], [35]–[38], in which both CD4^+^ [15], [18] and CD8^+^ [37], [39] cells are involved. The results presented in this paper thus reinforce the observation that these cells might be involved in the development of associated diseases.

Regulatory T (T_reg_) cells express CD4^+^ and CD25^+^ and have potent immune response suppressive activity [40]; however, their exact role in HTLV-1 infection is unknown. In recent studies, leukaemia cells from patients with ATLL strongly expressed FoxP3 [32], a specific marker for T_reg_ cells. These cells constitute about 2–3% of human CD4^+^ T cells [40], and they have increasingly been found to play conflicting roles in both autoimmune and infectious disease. Yamano et al. [41] showed that HTLV-1 *Tax* induced dysfunction of CD4^+^CD25^+^ T cells in patients with HTLV-I-associated neuroimmunological disease. More recently, Toulza et al. [42] proposed that CD4^+^CD25^high^ FoxP3^+^ cells are the chief determinants of the efficiency of T-cell-mediated immune control of HTLV-1. In our study, however, we observed no pathologic or clinical manifestations of inflammatory status. Thus, further physiopathologic characterization is required to define the association between natural STLV-1 infection and associated disease in this animal model. In humans, only 2–8% of HTLV-1-infected persons develop a severe HTLV-1-associated disease like ATLLL or TSP/HAM during their lifetime, generally after a long latency [26]. Furthermore, in monkeys, associated diseases such as ATLL have been related, in some cases, to co-infection with SIV [17], [43]. Mandrills are also naturally infected with simian immunodeficiency virus (SIVmnd) [44]. As only monkeys infected with STLV-1 were selected for the present study, co-infection with SIV in our mandrill colony might account for the development of associated diseases. Thus, long-term clinical studies in non-human primates are required to evaluate the relation between STLV-1 infection and the development of associated diseases.

In order to elucidate the role of the immune system in controlling STLV infection, we evaluated the frequency of circulating effector T cells against *Tax* in chronically STLV-1-infected monkeys. Although the response to the various peptides was different in each monkey, a response was detected in all. In our studies with chronically infected squirrel monkeys, we showed the presence of a cytolytic T-cell response against *Tax* in enriched CD8^+^ T cells [45]. The present study reports for the first time a cellular immune response against *Tax*, although we could not identify the cells involved, and further studies are needed.

To determine whether the cellular immune response during chronic STLV-1 infection in natural hosts controls STLV-1 replication over time, resulting in the long latency, immunosuppression studies with tacrolimus (FK506) were conducted. It has been reported previously that some graft recipients treated with immunosuppressive drugs develop ATLL [46]–[48]. In a study of patients treated with various immunosuppressive drugs, those given tacrolimus had a higher risk for ATL than those treated with cyclosporin [49], [50]. In our study, we observed no clinical manifestation related to STLV-1 infection after immunosuppression, although some significant modifications were observed in the activation markers and the proliferation status of CD4^+^ and CD8^+^ T cells in tacrolimus-treated monkeys. Most importantly, in the animal with the highest proviral load, an increased STLV-1 proviral load was observed after the immunosuppression period. In the same animal, the increase in proviral load correlated with increased numbers of activation and proliferation markers of CD4^+^ T cells and specifically with increased numbers of the activation marker HLADR^+^ in CD8^+^ T cells. This observation confirms the implication of T-cell expansion in viral replication and in the expansion of infected cells after immunosuppression. One limitation of this part of our study is the small number of animals in the two groups undergoing immunosuppression. More significant effects might be found in larger numbers of animals with higher proviral loads and more aggressive immunosuppression, which could lead to the development of HTLV-1-associated diseases such as ATLL. We are now focusing our efforts in this direction. For example, it has been reported that treatment with cyclophosphamide negatively affects the number of T_reg_ cells but totally conserves the other Th1 repertory cells [51]. Thus, to evaluate the role of T_reg_ cells in HTLV-1 pathogenesis, we shall investigate the effect of cyclophosphamide in STLV-1 infected monkeys.

In summary, we have demonstrated that naturally STLV-1-infected mandrills could be a suitable model for studying the relation between host and virus. Further studies are needed to determine whether the different compartments of the immune response during infection are responsible for the long latency, by controlling viral replication over time. Such studies would provide important information for the development of immune-based therapeutic strategies.

## Materials and Methods

### Animals

We studied 142 semi-free-ranging mandrills housed in the Primatology Centre of the CIRMF, Gabon. Mandrills were handled in accordance with standard operating procedures in the CIRMF as well as in accordance with the United States National Institutes of Health (NIH) guidelines for the Care and Use of Laboratory Animals. All the animal protocols and procedures were approved by the Ethical Committee of Ile de France for animal experimentation and by the Gabonese ethics committee for animal experimentation, and registered under No. 08-006. The primate centre has three veterinarians specialized in primates—an ethologist, a primatologist, and an ecologist—and all experiments were conducted under their supervision. Between November 2006 and January 2007, blood samples were collected from mandrills in EDTA-K2 tubes under ketamine-HCl (10 mg/kg body weight) anesthesia. For other immunovirological and immunosuppression studies (see below), six STLV-1-infected mandrills were selected from the free-ranging colony on the basis of their STLV-1 proviral load, two monkeys having a high load, two medium and two low. They were also selected on the basis of their ability to live together in an isolated enclosure without aggressive behavior and without disruption of the free-ranging breeding colony during the experimentation period.

### Serological screening

It has been reported previously that STLV-1 from mandrills is highly homologous to human HTLV-1 subtype B (see introduction). Thus, we used the criteria for diagnosis of HTLV-1 infection in humans to evaluate the presence of STLV-1 in our mandrill colony [11], [19].

Plasma was screened for antibodies to HTLV-I/II/STLV-1 with two enzyme-linked immunosorbent assays (ELISAs), namely HTLV-I Platelia New (Biorad, Marnes-la-Coquette, France) and Vironostica (Biomerieux, Marcy l'Etoile, France). STLV-1 infection was confirmed by western blotting (HTLV blot 2.4, Diagnostic Biotechnology Ltd, Singapore). Mandrills were considered STLV-1 positive when a complete western blot profile was obtained.

### Immunosuppression

Immunosuppression was induced in mandrills by administering tacrolimus (Prograf®, 5 mg/ml, Astellas Ireland, Cokerry, Ireland) via an intraperitoneally implanted osmotic pump (Alzet® Model 2ML4, Charles River Laboratories, l'Arbresle, France) at a dose of 0.1 mg/kg body weight per day for 28 days. Three mandrills received tacrolimus, and the other three received phosphate-buffered saline, pH 7.2 (Biomerieux, Marcy l'Etoile, France). Blood samples were collected at weeks –3, –2, –1, 0, +1, +2, +3, +4, +5, +6, +7, and +8.

Before immunosuppression of the infected monkeys, the baseline numbers of T-cell subsets (see below) were evaluated for each animal (Table S1). The monkeys were healthy, and there was no significant difference in the T-cell subsets of these STLV-1-infected monkeys and those of 59 uninfected animals (Table S1).

### Pharmacokinetics of tacrolimus

Tacrolimus concentrations were determined with Pro-Trac II Tacrolimus ELISA kits, as recommended by the manufacturer (DiaSorin, Stillwater, Minnesota, USA), in whole blood, before treatment and weekly for 6 weeks during treatment. Tacrolimus concentrations were determined by interpolation from a standard curve.

### Flow cytometric analysis of cell-surface and intracellular marker expression

Whole-blood samples were analyzed by four-color flow cytometry with a standard procedure and a panel of monoclonal antibodies (mAbs). The mAbs were originally designed to detect human molecules but cross-react with the mandrill counterparts. The mAbs were against CD4-fluorescein isothiocyanate (FITC) (clone MT4-77), CD4-phycoerythrin (PE) (clone L200), CD3-allophycocyanin (clone SP34-2), CD8-peridine chlorophyll protein (clone SK1), HLA DR-PE (clone G46-6), CD25-PE (clone 2A3), Ki67-FITC (clone B56), CD28-PE (clone L293), and CD95-FITC (clone DX2), all obtained from BD Bioscience (Le Pont de Claix, France). At least 10 000 events were acquired in the lymphocyte square on a FACScalibur flow cytometer driven by the CellQuest software package (Becton Dickinson, Heidelberg, Germany). Data were analyzed with FlowJo software v7.2 (Tree Star, Inc., Ashland, Oregon, USA).

### STLV proviral load

PBMCs were separated by Ficoll density gradient centrifugation. PBMC DNA was extracted by the phenol/chloroform procedure. STLV proviral load was quantified by using a real-time SYBR Green PCR method with IQ SYBR Green Supermix (Biorad, Marnes-la-Coquette, France) on an iCycler Thermal Cycler coupled with the iQ5 Optical System (Biorad, Marnes-la-Coquette, France). The primer sets SK43 (CGGATACCCAGTCTACGTGT) and SK44 (GAGCCGATAACGCGTCCATCG), at a final concentration of 1.5 µmol/l, were used to amplify a 159-base-pair fragment of the *Tax* gene (location, SK43:2–22; SK44:141–161). The reaction proceeded as follows: 5 min at 95°C, 50 cycles of 10 s at 95°C, 5 s at 60°C, and 10 s at 72°C. Melting-curve analysis was performed in 0.5°C increments from 60°C to 95°C. A standard curve was generated with 10-fold serial dilutions of MT4 cells, each of which is known to contain seven copies of proviral HTLV. To standardize the cell number, the albumin gene was also quantified by TaqMan PCR with iTaq Supermix and ROX (Biorad, Marnes-la-Coquette, France). The TaqMan probe ALBT (5′FAM-CCTGTCATGCCCACACAAATCTCTCC-TAMRA3′) and the primers ALBF (GCTGTCATCTCTTGTGGGCTGT) and ALBR (ACTCATGGGAGCTGCTGGTTC) were used as described elsewhere (Gabet et al., 2003). The PCR protocol consisted of 3 min at 95°C, 50 cycles for 10 s at 95°C, and 45 s at 60°C. The STLV proviral load is reported as the copy number per 100 cells.

### Enzyme-linked immunospot (ELISPOT) assay for IFN-γ

We used 82 overlapping peptides covering the entire Tax protein [52], which were divided into 17 pools, each containing five overlapping peptides of 12 amino acids (Table 2). The peptides were modified according to the Tax sequences obtained from an STLV-1 infected mandrill (GenBank Accession no. FJ755931). The peptides were diluted to 1 mg/ml in distilled water.

The ELISPOT assay was carried out as previously described [53]. Briefly, Multiscreen filtration plates (MAIPS4510, Millipore, Bedford, Massachusetts, USA) were coated with 15 µg/ml of an mAb against IFN-γ (1-D1K, Mabtech, Sweden) overnight at 4°C. The plates were then washed five times with RPMI-1640 medium and blocked with RPMI 1640 supplemented with L-glu, pen/strep, and 10% fetal calf serum for 2 h. Then, 2×10^5^ PBMC in the above medium were added in a total volume of 200 µl, in the presence or absence of 10 µg/ml of relevant peptide, and incubated for 40 h at 37°C. The cells were then incubated with 100 µl of 1 µg/ml biotinylated mAb 7-B6-1 against IFNγ (Mabtech, Sweden) for 3 h at room temperature. After washing, the substrate BCIP/NBT (Kirkegaard and Perry Labs, Gaithersburg, Maryland, USA) was added, and the solution was incubated in the dark for 10–20 min until the appearance of dark-blue spots. The reaction was stopped by washing with cold tap-water, and the spots were counted with an Immunospot Image Analyzer (Zeiss, Oberkochen, Germany). The frequency of responding cells, obtained after subtracting background spots in negative control wells (medium alone), was expressed as spot-forming cells per million PBMC.

### Statistical analysis

The Mann-Whitney U test was used to compare groups. Correlations were sought by using the standard Pearson correlation coefficient or Spearman's rank correlation test. Significance was assumed at *p*<0.05. All analyses were performed with Statistica software v7.1. (StatSoft France, www.statsoft.fr).

## Supporting Information

Table S1 Virological and immunological baseline values for the six STLV-1 infected mandrills included in the Elispot assay and in the immunosuppression treatment and for 59 uninfected animals.
(0.05 MB DOC)

Figure S1 Correlation between age and numbers of CD4+ and CD8+ T cells in STLV-infected and uninfected mandrills. (A) Absolute numbers of CD4+ T cells (cells/µl). (B) Absolute numbers of CD8+ T cells (cells/µl). Regression curves are shown as unbroken lines for STLV-infected mandrills and as dotted lines for uninfected mandrills.
(0.06 MB TIF)
