# Supplementary material for: Dynamic Interaction between STLV-1 Proviral Load and T-Cell Response during Chronic Infection and after Immunosuppression in Non-Human Primates
Source: PLoS One. 2009 Jun 25;4(6):e6050. doi: 10.1371/journal.pone.0006050 (PMC2698465; doi:10.1371/journal.pone.0006050)
Supplement: Table S1 — Virological and immunological baseline values for the six STLV-1 infected mandrills included in the Elispot assay and in the immunosuppression treatment and for 59 uninfected animals. (0.05 MB DOC) [file pone.0006050.s001.doc]

**Table S1.**

| Variable | STLV-1 infected mandrills | | | | | | STLV-1  *n* = 6  (mean ± SD) | Uninfected  *n* = 59  (mean ± SD) |
| --- | --- | --- | --- | --- | --- | --- | --- | --- |
|  | mnd1 | mnd2 | mnd3 | mnd4 | mnd5 | mnd6 |  |  |
| Sex | F | F | F | F | M | F |  |  |
| Age (years) | 12 | 10 | 7 | 6 | 7 | 19 | 10.2 ± 4.9 | 10.6 ± 6.5 |
|  |  |  |  |  |  |  |  |  |
| Lymphocyte subset: |  |  |  |  |  |  |  |  |
| CD4^+^ T cells (number/mm^3^) | 593 | 563 | 741 | 351 | 586 | 185 | 503 ± 199 | 476 ± 315 |
| CD8^+^ T cells (number/mm^3^) | 769 | 794 | 1242 | 611 | 1217 | 619 | 875± 284 | 589 ± 353 |
| CD4^+^/CD8^+^ T cells (ratio) | 0.8 | 0.7 | 0.6 | 0.6 | 0.5 | 0.3 | 0.6 ± 0.2 | 0.8 ± 0.4 |
| % HLADR^+^ in CD4^+^ T cells | 1.9 | 7.6 | 7.6 | 1.1 | 3.8 | 6.6 | 4.8 ± 2.9 | 4.2 ± 2.1 |
| % HLADR^+^ in CD8^+^ T cells | 4.9 | 9.6 | 7.9 | 3.7 | 4.1 | 6.8 | 6.2 ± 2.3 | 8.2 ± 4.2 |
| % CD25^+^ in CD4^+^ T cells | 12.6 | 14.1 | 23.1 | 13.3 | 15.9 | 30.8 | 18.3 ± 7.2 | 20.9 ± 8.2 |
| % CD25^+^ in CD8^+^ T cells | 9.7 | 12.1 | 8.1 | 5.9 | 6.1 | 13.4 | 9.2 ± 3.1 | 9.6 ± 5.4 |
| % Ki67^+^ in CD4^+^ T cells | 2.6 | 3.1 | 2.9 | 1.9 | 2.6 | 2.4 | 2.6 ± 0.4 | 4.3 ± 2 |
| % Ki67^+^ in CD8^+^ T cells | 2.4 | 2.1 | 2.3 | 1.4 | 1.5 | 1.3 | 1.8 ± 0.5 | 3.6 ± 2.4 |
|  |  |  |  |  |  |  |  |  |
| STLV-1 proviral load in PBMC (%) | 0.9 | 2.2 | 4.5 | 0.2 | 1.30 | 5.4 | – | – |

SD: standard deviation.
