# Supplementary figures and images for: Dynamic Interaction between STLV-1 Proviral Load and T-Cell Response during Chronic Infection and after Immunosuppression in Non-Human Primates
Source: PLoS One. 2009 Jun 25;4(6):e6050. doi: 10.1371/journal.pone.0006050 (PMC2698465; doi:10.1371/journal.pone.0006050)

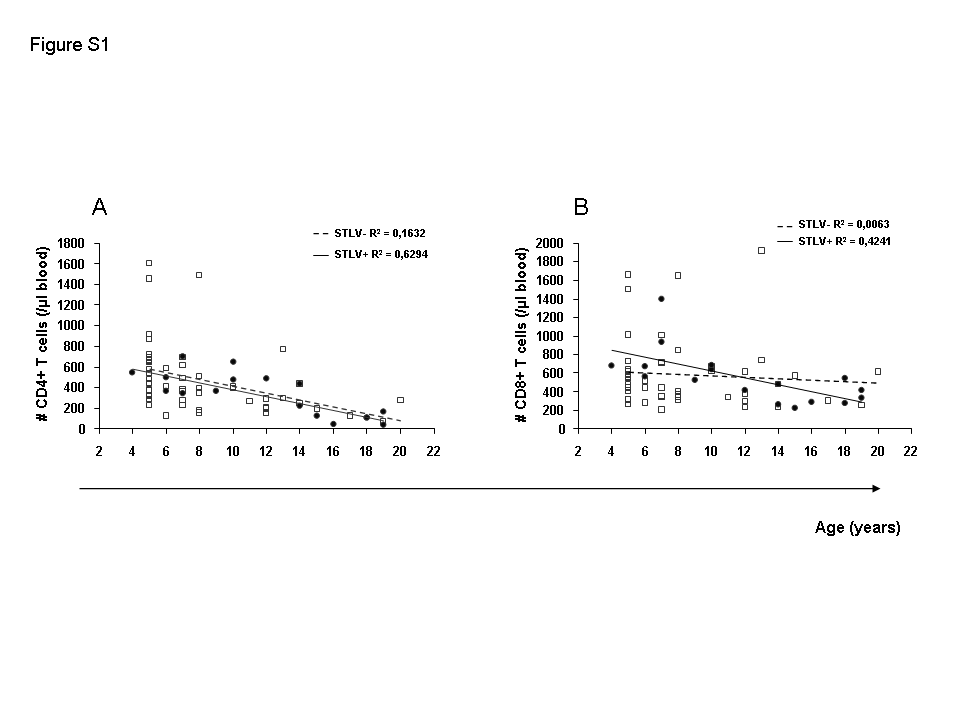

Supplement: Figure S1 — Correlation between age and numbers of CD4+ and CD8+ T cells in STLV-infected and uninfected mandrills. (A) Absolute numbers of CD4+ T cells (cells/µl). (B) Absolute numbers of CD8+ T cells (cells/µl). Regression curves are shown as unbroken lines for STLV-infected mandrills and as dotted lines for uninfected mandrills. (0.06 MB TIF) [file pone.0006050.s002.tif]
